# Bacteriophage preparation lytic for *Shigella* significantly reduces *Shigella sonnei* contamination in various foods

**DOI:** 10.1371/journal.pone.0175256

**Published:** 2017-03-31

**Authors:** Nitzan Soffer, Joelle Woolston, Manrong Li, Chythanya Das, Alexander Sulakvelidze

**Affiliations:** Intralytix, Inc., Baltimore, Maryland, United States of America; University of Helsinki, FINLAND

## Abstract

ShigaShield^™^ is a phage preparation composed of five lytic bacteriophages that specifically target pathogenic *Shigella* species found in contaminated waters and foods. In this study, we examined the efficacy of various doses (9x10^5^-9x10^7^ PFU/g) of ShigaShield^™^ in removing experimentally added *Shigella* on deli meat, smoked salmon, pre-cooked chicken, lettuce, melon and yogurt. The highest dose (2x10^7^ or 9x10^7^ PFU/g) of ShigaShield^™^ applied to each food type resulted in at least 1 log (90%) reduction of *Shigella* in all the food types. There was significant (P<0.01) reduction in the *Shigella* levels in all phage treated foods compared to controls, except for the lowest phage dose (9x10^5^ PFU/g) on melon where reduction was only ca. 45% (0.25 log). The genomes of each component phage in the cocktail were fully sequenced and analyzed, and they were found not to contain any “undesirable genes” including those listed in the US Code for Federal Regulations (40 CFR Ch1). Our data suggest that ShigaShield^™^ (and similar phage preparations with potent lytic activity against *Shigella* spp.) may offer a safe and effective approach for reducing the levels of *Shigella* in various foods that may be contaminated with the bacterium.

## Introduction

*Shigella* is an important cause of morbidity and mortality throughout the world, causing approximately 125 million *Shigella* infections / year and an estimated 14,000 deaths, mostly among children <5 years of age [1,2]. In the United States, *Shigella* is the third most common causes of gastroenteritis, with at least 500,000 cases of shigellosis linked diarrheal events in the USA annually [3,4]. General symptoms of shigellosis include bloody watery diarrhea, fever, nausea, and tenesmus (pain in the bowel), while complications include post-infection arthritis, sepsis, seizures (in young children) and hemolytic-uremic syndrome [4]. The two main routes of transmission include (i) through contaminated foods, and (ii) water contaminated with human waste; *Shigella* are easily transmitted through human contact due to its low infectious dose of 10–200 cells [5]. *Shigella* have been isolated from almost all food types, including salads (potato, tuna, shrimp, macaroni, or chicken), fresh fruits and vegetables, poultry, milk and dairy products, deli meats, and seafood [6]. In the USA, the main *Shigella* species isolated from contaminated foods is *Shigella sonnei* (found in ca. 80% of tested foods during 1998–2008). Internationally, *Shigella flexineri* (66%) and then *Shigella sonnei* (18%) are the most common food contaminant *Shigella* species [6,7]. The “ready-to-eat” foods (i.e., foods that are ready to be eaten, without any additional processing) are of particular concern because of the lack of the heat processing step (e.g., cooking) that could kill *Shigella* and render those foods safer to eat. *Shigella* also present a major concern to military personnel and travelers, especially when the deployment and/or travel is in the endemic areas where commercial food sanitation standards are poor and enforcement of those standards is lenient [8]. For example, several studies have shown a considerable loss of person-hours because of traveler’s diarrhea among U.S. military personnel deployed to the Persian Gulf during operations Desert Shield and Desert Storm, well as during peacetime operations [9–12]. The problem is further exacerbated by the increase of *Shigella* strains that are resistant to many commonly available antibiotics. For example, in 2013, the Centers for Disease Control and Prevention (CDC) declared antibiotic-resistant *Shigella* an urgent threat in the United States [13]. Also, an increasing prevalence of antibiotic resistance has been recently reported in the USA for the *Shigella* strains transmitted through sexual contact among men (nearly a quarter of *Shigella* isolates tested in New York City showed decreased susceptibility or resistance to recommended antibiotics) [14]. The prevalence of antibiotic-resistant *Shigella* strains also appears to be on the rise in foods; for example, in a recent study where more than 1,600 food samples (seafood, fresh vegetables, and meats) were examined, 89% of the *Shigella* strains were found to be multidrug resistant [6]. In these settings, novel non-antibiotic approaches are required to reduce the prevalence and levels of *Shigella* in various foods, which may help reduce the risk of shigellosis in the civilian (including young children) and military (including in US troops stationed in developing countries) populace. Lytic bacteriophages may provide one such relatively novel, environmentally friendly “green” approach.

Bacteriophages are bacterial viruses that are the most abundant biological entities in the world, in one ml of seawater there is an estimated 10^7^ phages, with approximately 10^30^–10^31^ in the world [15]. Lytic phages have a potent, highly specific bactericidal activity against their targeted bacterial cells–a feature that enables a targeted killing of specific problem-causing bacteria in various settings, without disturbing the normal–and often beneficial–microflora. Various bacteriophage-based food additives have been approved by the FDA for food safety applications, including (listed in chronological order of receipt of regulatory approval in the USA): ListShield^™^, Listex P-100^™^, EcoShield^™^, SalmoFresh^™^ and Salmonelex^™^ [16]. Here, we report the results of studies in which the ability of ShigaShield^™^ to reduce *Shigella* levels was evaluated in various foods experimentally contaminated with a *S*. *sonnei* strain. Various *Shigella* phage preparations have been extensively used therapeutically previously to prevent and/or treat shigellosis in humans [17] reviewed by [18]; however, to the best of our knowledge, there is only one previous report of successful use of a *Shigella* phage preparation in food safety applications [19]. The phage preparation described in this communication (i.e., ShigaShield^™^) is currently undergoing FDA and USDA review for the GRAS (Generally Recognized As Safe) status (GRN 672).

## Materials and methods

### Bacteriophage preparation

ShigaShield^™^ is a bacteriophage “cocktail” essentially identical to the previously-described ShigActive^™^ preparation [20]. It is composed of 5 lytic phages (mixed in approximately equal concentrations): SHSML-52-1 (ATCC PTA-121241), SHFML-11 (ATCC PTA-121234), SHSML-45 (ATCC PTA-121238), SHFML-26 (ATCC PTA-121236), and SHBML-50-1 (ATCC PTA-121239). The same lot (# 1112I210158) was used in all studies. The phage preparation was supplied in normal saline solution (0.1 M NaCl, pH 6.5–7.5), and was stored refrigerated (2–8°C) until use. An additional 36 strains were examined for their susceptibility to ShigaShield^™^ employing the same method used on the other *Shigella* strains in our previous study [20].

### Genomic sequencing of component bacteriophages

Each phage included in ShigaShield^™^ was sequenced, annotated, and the sequences were deposited in GenBank at the National Center for Biotechnology Information (NCBI) (Accession numbers: KX130865.1; KX130864.1; KX130863.1; KX130862.1; and KX130861.1). Briefly, each monophage was sequenced on a MiSeq (Illumina, San Diego, CA), assembled and annotated on the CLC Bio Genomic Workbench software, using default settings (version 7.0.2; CLC Bio, Cambridge, MA) at the BioAnalytical Laboratory of the Institute of Marine and Environmental Technology (IMET). Annotations were confirmed using two additional annotation pipelines: Rapid Annotation Subsystem Technology (RAST, version 4.0) and the Phage Annotations Using Subsystems Technology server (PHAST, version 1.0). Lastly, each genome was scanned for the “undesirable genes” listed in 40 CFR §725.421, and for any potential virulence factors on the Virulence BLAST Interface (VBI) using default parameters.

### Food items

The ability of ShigaShield^™^ to reduce the levels of *Shigella* in foods was examined in (1) smoked salmon, (2) pre-cooked chicken breast strips, (3) sliced deli corned beef, (4) honeydew melon, pre-cut and packaged, (5) 1.5% vanilla flavored yogurt, and (6) long leaf lettuce packaged in a bag. These foods were selected to encompass a variety of viscosities, carbon/protein levels, and textures. All food items were purchased in local Baltimore grocery stores and were not washed, heated, or otherwise pre-treated prior to the studies.

### Bacterial strain used to experimentally contaminate foods

A nalidixic acid resistant strain of *Shigella sonnei* was selected by serially passaging the Intralytix SH.s43 (original identification University of Maryland-Pakistan Isolate 90) on Luria-Bertani (LB) (Neogen, MI) agar plates supplemented with increasing concentrations of nalidixic acid (Arcos Organics, NJ). The strain underwent ≤8 serial passages before it was determined to be nalidixic acid-resistant at a concentration of 25 μg/ml. After the serial passages, the strain was assigned an Intralytix designation SH.s53. It is susceptible to all phages in ShigaShield^™^. The strain was stored at –80°C in 70% LB broth/30% glycerol supplemented with 25 μg of nalidixic acid/mL. For each study, a frozen aliquot of SH.s53 was thawed and grown (37 ± 2°C, 16–24 h) in LB broth supplemented with nalidixic acid (25 μg/ml). Overnight growth for this strain corresponds to ca. 2x10^8^ Colony Forming Units per mL (CFU/mL). An overnight culture of SH.s53 was applied to all foods in approximately same concentrations, ranging from ca. 2x10^3^ CFU/g (melon, chicken, beef deli and yogurt) to ca. 3x10^3^ CFU/g (lettuce) to ca. 4x10^3^ CFU/g (salmon). The bacteria was evenly spread across the surfaces of or mixed into each food item using a hockey stick. The bacteria-contaminated samples then rested in room temperature for 60 minutes before ShigaShield^™^ application.

### ShigaShield^™^ application

Immediately prior to use, ShigaShield^™^ was removed from refrigerated storage and diluted in clean tap water as necessary. ShigaShield^™^ and controls were applied using a Basic Spray Gun Model #250 (Badger Air-Brush Co., Franklin Park, IL) to evenly spray the treatment onto all food surfaces, except the yogurt, where the ShigaShield^™^ or water treatments were mixed into the food. Each food item, except lettuce, was treated with three levels of ShigaShield^™^ or water. All treatments were 0.9mL per 100g of food, the same volume of water was applied for the control. Three concentrations (in Plaque Forming Units, or PFU) of ShigaShield^™^ (1x10^10^ PFU/mL, 1x10^9^ PFU/mL, or 1x10^8^ PFU/mL) were used to obtain a final concentration of ca. 9x10^7^ PFU/g, 9x10^6^ PFU/g, or 9x10^5^ PFU/g on the foods, respectively. Lettuce was treated with two levels of ShigaShield^™^ or PBS, applied at 1mL per 100g. Two concentrations of ShigaShield^™^ (2x10^9^ PFU/mL or 2x10^8^ PFU/mL) were used to obtain a final concentration of ca. 2 x10^7^ PFU/g or 2x10^6^ PFU/g on the lettuce, respectively.

### General design of efficacy studies in foods

After treatment, food samples were incubated at room temperature for 5 minutes. Three replicates of 25 g sample sizes from each food type and experimental group were placed into sterile bags, and 225 mL of sterile peptone water (Becton, Dickinson and Co., MD) was added. The bags were hand mushed briefly and stomached for a minimum of 30 seconds. The number of viable *Shigella* in the samples was determined by plating 0.5 mL aliquots of the stomached food/peptone water mixture onto separate MacConkey plates (Becton, Dickinson and Co., MD) supplemented with nalidixic acid (25 mg/mL). The plates were incubated (35 ± 2°C, 24±2 hr), and the final bacterial amounts (CFU/g) were calculated after counting the colonies as follows:

$$\frac{Total\;CFU}{g\;of\;treated\;food}=\frac{CFU}{0.5\;mL\;\;plating}*\frac{225\;mL\;peptone}{25\;g\;sample}$$

### Evaluation of results and statistical analysis

The efficacy of ShigaShield^™^ in reducing the levels of *Shigella* in various foods was determined by comparing the levels of *Shigella* recovered from the foods treated with phage preparation vs. controls. Statistical analysis was performed using the Analysis of Variance (ANOVA) test for each food type independently. Tukey- Kramer multiple comparison post-hoc tests were done to determine which phage concentrations were significantly different when significance was determined via ANOVA (P<0.01). All statistical analysis was performed using version 3.05 of GraphPad InStat and version 4.0 of GraphPad Prism (GraphPad Software, San Diego, CA; www.graphpad.com).

## Results and discussion

### Phage host range

In a previous study by Mai and colleagues, this phage cocktail was shown to lyse 62 of 65 *Shigella* strains representing all four known species of *Shigella*: *S*. *flexneri*, *S*. *dysenteriae*, *S*. *sonnei* and *S*. *boydii* [20] Since that publication, the cocktail was tested for its activity against an additional 36 multidrug-resistant S*higella* strains obtained from the Centers for Disease Control and Prevention (CDC). This additional collection of *Shigella* included 5 *S*. *boydii*, 4 *S*. *dysenteriae*, 8 *S*. *flexneri*, and 19 *S*. *sonnei* strains (See Table 1). All new strains were resistant to at least 3 of the antibiotics tested by the National Antimicrobial Resistance Monitoring System (NARMS) (Nancy Strockbine, personal communication). All new multidrug-resistant *Shigella* strains were susceptible to the ShigaShield^™^ phage preparation when tested in the standard concentration of ca. 1x10^9^ PFU/mL. When these data are combined with the previous data reported by Mai and colleagues [20], the ShigaShield^™^ phage cocktail lysed 98 (97%) of the 101 *Shigella* strains in our collection. [20].

**Table 1:** List of new *Shigella* isolates tested for susceptibility to ShigaShield^™^.

Original ID	Intralytix ID	Provider	Shigella Serotype
2013C-3160	SH.f71	CDC, Atlanta GA	*flexneri*
2013C-3473	SH.s72	CDC, Atlanta GA	*sonnei*
2013C-3606	SH.f73	CDC, Atlanta GA	*flexneri*
2013C-3787	SH.f74	CDC, Atlanta GA	*flexneri*
2013C-4189	SH.f75	CDC, Atlanta GA	*flexneri*
2014C-3799	SH.s76	CDC, Atlanta GA	*sonnei*
2015C-3053	SH.s77	CDC, Atlanta GA	*sonnei*
2015C-3237	SH.s78	CDC, Atlanta GA	*sonnei*
2015C-3288	SH.s79	CDC, Atlanta GA	*sonnei*
2015C-3306	SH.s80	CDC, Atlanta GA	*sonnei*
2015C-3349	SH.s81	CDC, Atlanta GA	*sonnei*
2015C-3626	SH.s82	CDC, Atlanta GA	*sonnei*
2015C-3627	SH.s83	CDC, Atlanta GA	*sonnei*
2015C-3802	SH.s84	CDC, Atlanta GA	*sonnei*
2015C-3811	SH.s85	CDC, Atlanta GA	*sonnei*
2015C-4077	SH.s86	CDC, Atlanta GA	*sonnei*
2015C-4287	SH.s87	CDC, Atlanta GA	*sonnei*
2015C-4463	SH.s88	CDC, Atlanta GA	*sonnei*
2015C-4465	SH.s89	CDC, Atlanta GA	*sonnei*
2015C-4836	SH.f90	CDC, Atlanta GA	*flexneri*
2015C-5216	SH.s91	CDC, Atlanta GA	*sonnei*
2016C-3073	SH.f92	CDC, Atlanta GA	*flexneri*
2016C-3082	SH.s93	CDC, Atlanta GA	*sonnei*
2016C-3328	SH.s94	CDC, Atlanta GA	*sonnei*
2016C-3355	SH.f95	CDC, Atlanta GA	*flexneri*
2016C-3375	SH.f96	CDC, Atlanta GA	*flexneri*
2013AM-2809	SH.d97	CDC, Atlanta GA	*dysenteriae*
2014AM-1029	SH.b98	CDC, Atlanta GA	*boydii*
AM11413	SH.d99	CDC, Atlanta GA	*dysenteriae*
AM17886	SH.d100	CDC, Atlanta GA	*dysenteriae*
AM22438	SH.b101	CDC, Atlanta GA	*boydii*
AM25896	SH.d102	CDC, Atlanta GA	*dysenteriae*
AM38301	SH.b103	CDC, Atlanta GA	*boydii*
AM41657	SH.b104	CDC, Atlanta GA	*boydii*
AM49802	SH.b105	CDC, Atlanta GA	*boydii*

All strain listed are susceptible to ShigaShield^™^. These strains are in addition to the 62 strains previously analyzed for susceptibility [20]

### Phage genome analysis

Each phage genome in ShigaShield^™^ was fully sequenced and annotated to determine whether there were any potentially "undesirable" genes (e.g., virulent and/or toxic genes) present. No toxin, virulence, repressor genes, integrases, recombinases nor any bacterial gene listed in the US Code for Federal Regulations (40 CFR §725.421) were detected. The absence of these "undesirable" genes has important safety implications. In this context, the physiological safety of ShigaShield^™^ (under the name ShigActive^™^) has been previously demonstrated in mice that were administered oral doses at both the recommend upper limit dosage (ca. 1x 10^8^ PFU/g) and a 10-fold higher dosage (1x10^9^ PFU/g) [20]. In that study, no physiological signs of toxicity were detected even with the highest dose of ShigActive^™^. Moreover, the metagenomic analysis showed that oral administration of bacteriophage preparation (in contrast to an antibiotic) elicited no changes in the overall microbiome of the mice (i.e., non-targeted bacteria were not affected) [20].

The genome analyses data presented in this manuscript provide further supporting evidence of the safety of these bacteriophages from the genomic composition standpoint. Namely, the genomic data suggest that the component bacteriophages are lytic phages with no "undesirable" genes in their genomes (and thus no evidence of any potentially dangerous transducing ability), are safe, and well suited for biocontrol applications.

### Efficacy of ShigaShield^™^ on foods experimentally contaminated with *Shigella*

#### Lettuce

**Fig 1:**
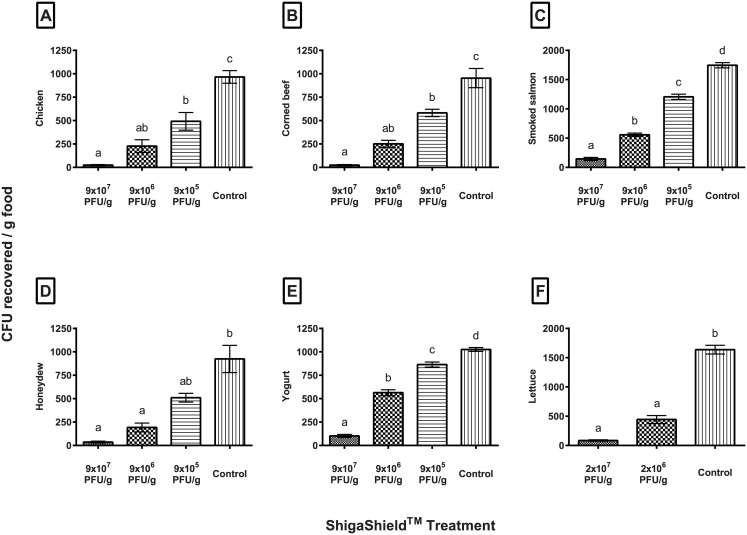
Effect of ShigaShield on the *Shigella* levels in various foods. Mean CFU recovered per gram of food (±SEM); for each food, means with different letters are significantly different (*P*<0.01). P-values are based on Tukey-Kramer multiple comparison post-hoc tests.

Contaminated lettuce has been the cause of several major foodborne *Shigella* outbreaks [21]. In this study, lettuce was the first food type tested with ShigaShield^™^. Because lettuce was the preliminary testing food, some of the methods were slightly different compared to the other foods examined. For example, PBS instead of water was used as a control, and different ShigaShield^™^ doses (i.e., the highest dose on lettuce was 2x10^7^ vs. 9x10^7^ PFU/g in the other studies). Even with the comparatively lower phage dosages in lettuce vs. the other studies, the higher phage application (2x10^7^ PFU/g) still reduced *Shigella* levels by ca.1.3 log (95%) (Fig 1-F, Table 2). The lower dosage tested (2x10^6^ PFU/g) also performed well, with 73% reduction or 0.6 log reduction in *Shigella*. Both doses were significantly different from the PBS controls (P<0.001) (Fig 1-F, Table 2), but not significantly different from each other, indicating that the lower and upper doses worked statistically similarly. This suggests that slight variations in application rates (e.g., due to human error or minor modifications in treatment protocols among various food producers) are not likely to significantly alter the efficacy.

**Table 2:** Reduction in the *Shigella* levels in various foods treated with bacteriophages vs. water- or PBS-treated controls.

Food	Group (phage concentration)	% Reduction±SEM	Log Reduction±SEM
**Lettuce**	A*	95±0.73	1.3±0.06
	B*	73±4.13	0.6±0.07
	D*	0±4.58	0±0.02
**Salmon**	A	92±2.73	1.098±0.08
	B	68±2.73	0.50±0.02
	C	31±4.49	0.16±0.02
	D	0±4.49	0±0.01
**Chicken**	A	98±0.62	1.6±0.10
	B	76±6.94	0.7±0.15
	C	49±9.73	0.3±0.08
	D	0±6.94	0±0.03
**Corned beef**	A	97±0.63	1.6±0.10
	B	74±3.93	0.6±0.11
	C	39±4.12	0.2±0.10
	D	0±10.73	0±0.049
**Yogurt**	A	90±1.55	1.01±0.07
	B	45±3.09	0.26±0.03
	C	16±2.68	0.07±0.01
	D	0±2.026	0±0.01
**Melon**	A	96±1.95	1.44±0.14
	B	79±8.78	0.7±0.12
	C	45±8.78	0.25±0.04
	D	0±27.30	0±0.07

Mean and Standard Error of Mean are shown (SEM). Group A* = 2x10^7^ PFU/g; Group B* = 2x10^6^ PFU/g; Group D* = PBS control; Group A = 9x10^7^ PFU/g; Group B = 9x10^6^ PFU/g; Group C = 9x10^5^ PFU/g; Group D = water control.

#### Smoked salmon

After treatment regimens and doses were established using lettuce, additional foods were treated with ShigaShield^™^, starting with smoked salmon. Treatment with all doses of ShigaShield^™^ resulted in statistically significant reduction in the *Shigella* levels compared to water controls (ANOVA P<0.0001; post-hoc tests = P<0.001 for all) (Table 2). As expected, the lowest dose was less effective at reducing *Shigella* contamination compared to the higher doses (31% vs 68% and 92%). There was ≥ 1 log reduction in the *Shigella* levels with 9x10^7^ PFU/g in all the tested replicates (Fig 1-C, Table 2). These data are in general agreement with the reduction of *Listeria monocytogenes* observed in smoked salmon samples treated with *L*. *monocytogenes*-specific phage preparation ListShield^™^ [22].

#### Precooked chicken breast strips

When ShigaShield^™^ was applied onto precooked chicken breast strips at the dose of 9x10^7^ PFU/g, it significantly reduced *Shigella* levels by 98% (1.6 log) compared to the water control (P<0.001). This reduction is somewhat lower than that reported previously for other *Shigella* phages applied onto pre-cooked chicken samples [19]. In that study, phages were found to reduce the levels of *Shigella* by ca. 2.0 log after 48 hours. Two factors may be responsible for this difference, first, in our study, the contact time for phages was limited to 5 minutes vs. 48 h in the study by Zhang and colleagues [19], and longer incubation time in theory may increase phage treatment efficacy. Secondly, and perhaps more importantly, they [19] used a larger concentration of phages compared to our study (3x10^8^ PFU/g in their study vs. max. of 9x10^7^ PFU/g in our study). The efficacy of phage treatment is concentration-dependent, and using more phage is likely to yield a better reduction of the targeted bacterium’s level [23,24]. Our studies further support this idea. Namely, we observed less reduction in the *Shigella* levels with the lowest dose of ShigaShield^™^ (9x10^5^ PFU/g) compared to the highest dose (P<0.01) (Fig 1-A, Table 2). The difference between various doses fades away when the doses differ by less than 1 log. For example, the highest dose of 9x10^7^ reduced the targeted bacteria by 98% (1.6 logs), which is not significantly different (P>0.01) from the medium dose of 9x10^6^, which resulted in ~76% reduction (0.7 log). As noted earlier, this range of dose variation vs. efficacy may be important to ensure the efficacy of the phage applications in the real-life commercial settings, when slight variations in application rates (e.g., due to minor deviations in treatment protocols among various food producers) may be encountered.

#### Corned beef deli meat

ShigaShield^™^ application was also effective in reducing the levels of *Shigella* in corned beef samples, at all concentrations examined (p<0.001). Among the different doses, the highest dosage resulted in 97% (1.6 log) reduction of *Shigella* compared to 74% (0.6 log) resulting from the next dose of 9x10^6^ PFU/g (Fig 1-B, Table 2). All three treatments had significantly reduced CFU/g compared to water controls (P<0.001 for the concentrations 9x10^7^, 9x10^6^ PFU/g and P<0.01 for group 9x10^5^ PFU/g -Table 2). There was no significance (P>0.01) between group 9x10^7^ and 9x10^6^, or 9x10^6^ and 9x10^5^, however 9x10^7^ and 9x10^5^ PFU/g were significantly different (P<0.001) (Fig 1-C). The lowest dose group 9x10^5^ PFU/g had 39% reduction compared to water controls and 0.2 log reduction.

#### Yogurt

ShigaShield^™^ reduced the levels of *Shigella* in yogurt by ca. 1 log (90%) when the highest dose (9x10^7^ PFU/g) was used. There was a significant difference among the various treatment groups (P< 0.001), and all treatments were statistically different from each other (P<0.001) and from water controls (group 9x10^7^ and 9x10^6^, P<0.001, group 9x10^5^ PFU/g, P<0.01- Table 2, Fig 1-E). Interestingly, the efficacy of phage treatment declined more rapidly in yogurt compared to the other foods when lower concentrations of phage were used for treatment. For example, we observed only a 0.07 log (16%) reduction in the *Shigella* levels for the lowest dose (9x10^5^ PFU/g) of ShigaShield^™^ vs., for example, reduction of 0.2 log (39%) in corned beef samples with the similar low dose. Various factors may have contributed to this outcome. For example, in the yogurt experiment, the same PFU/g was applied as other foods, but phages were mixed inside the yogurt with a greater overall surface area compared to the other foods, which have likely resulted in fewer contacts between phages and their targeted bacterial cells. Another (or additional) possibility is that some yogurt ingredient(s) inhibited phages or provided additional layer of protection for the targeted bacterial cells so that larger phage concentrations were required to effectively lyse the bacteria. Additional studies will be required to determine the underlying mechanisms, but our studies show that with the proper phage concentration, ShigaShield^™^ can provide a significant reduction in *Shigella* levels even in such complex food matrices as is yogurt.

#### Honeydew melon

ShigaShield^™^ application significantly reduced the levels of *Shigella* in honeydew melon samples at all concentrations examined. The two higher doses (9x10^7^ PFU/g and 9x10^6^ PFU/g) both resulted in significant reduction in the *Shigella* levels compared to water controls (P<0.001), ranging from 0.7 to 1.44 log reductions, respectively (Table 2, Fig 1-D). While the lowest dose of 9x10^5^ did not significantly reduce *Shigella*, there was still a respectable 45% (0.25 log) reduction of *Shigella* compared to the water controls (Table 2, Fig 1-D). There was no significant difference among the 9x10^6^ PFU/g dose and either the higher or lower dosage; the middle range dose reduced *Shigella* by 79% (0.7 log) (Fig 1-D).

## Conclusion

Our studies continue to support the idea that lytic bacteriophages can be used to effectively reduce the levels of various foodborne bacteria in various foods, thus rendering those foods safer for human consumption. Although major foodborne outbreaks of *Shigella* infections are relatively rare in the United States, *Shigella* spp. do cause ca. 500,000 cases of illness in the USA annually [3,4,25]. The problem of shigellosis was recently highlighted by an outbreak in Flint, Michigan during September—October 2016, where the population was afraid to use water for handwashing due to the concerns of the water being contaminated with lead [26]. Although the role of contaminated foods in the outbreak has not been firmly established, it is likely that they played at least some—and possibly major—role in spreading the disease. Another, more direct example of foodborne shigellosis outbreak is the 406-person antibiotic resistant *S*. *sonnei* outbreak in 2000, which was linked to ready to eat dip [27]. ShigaShield^™^ and similar phage preparations lytic for *Shigella* may help reduce the incidence or severity of such outbreaks. Another important area of potential application is the use of phages to improve the safety of foods for the US military and/or travelers; e.g., for treating fresh fruits and vegetables in high risk *Shigella* locations overseas, where US troops are stationed but where the local sanitation standards and/or quality of water are not optimal, thus creating an increased risk of *Shigella* contamination.

The use of phage preparations for food safety applications has been gradually gaining traction in the United States, where several phage preparations targeting major foodborne pathogens like *L*. *monocytogenes*, *E*. *coli* O157:H7, and *Salmonella* are currently on the market. These preparations offer safe and effective intervention modalities for removing their targeted bacteria from various foods, without altering organoleptic qualities of those foods and their normal microflora / nutritional characteristics [16]. After ShigaShield^™^ becomes commercially available, it will be another addition to that growing family of all natural lytic phage preparations that can serve as an important additional tool for reducing the levels of *Shigella* in our foods and making them safer to eat.
